# Supplementary material for: Assessment of Prognostic Value of Cystic Features in Glioblastoma Relative to Sex and Treatment With Standard-of-Care
Source: Front Oncol. 2020 Nov 16;10:580750. doi: 10.3389/fonc.2020.580750 (PMC7705378; doi:10.3389/fonc.2020.580750)
Supplement: Supplementary file 1 [file Data_Sheet_1.PDF]

Supplement - Assessment of Prognostic Value of Cystic Features in GBM Relative to Sex and Treatment with Standard-of-Care

SUPPLEMENT 1: Known received treatments between cystic and noncystic patients

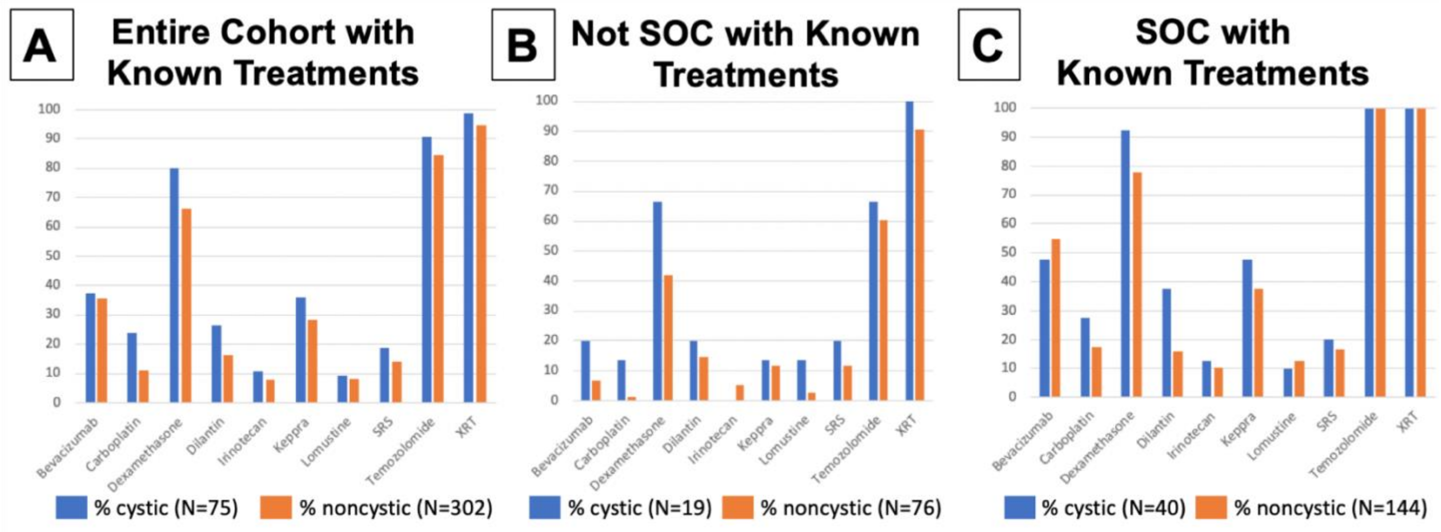

Visual comparison of the 10 most common known treatments received by patients with cystic GBM and patients with noncystic GBM across the whole cohort **(A)**, within patients marked as not having received the current SOC **(B)** and patients who did receive the current SOC **(C)**. Treatments were not significantly different in chi-square analysis for the whole cohort (p=0.5015). We did not have enough statistical power to satisfy the conditions necessary for a chi-square analysis in the other cases.

SUPPLEMENT 2: Matched cohorts for treatment

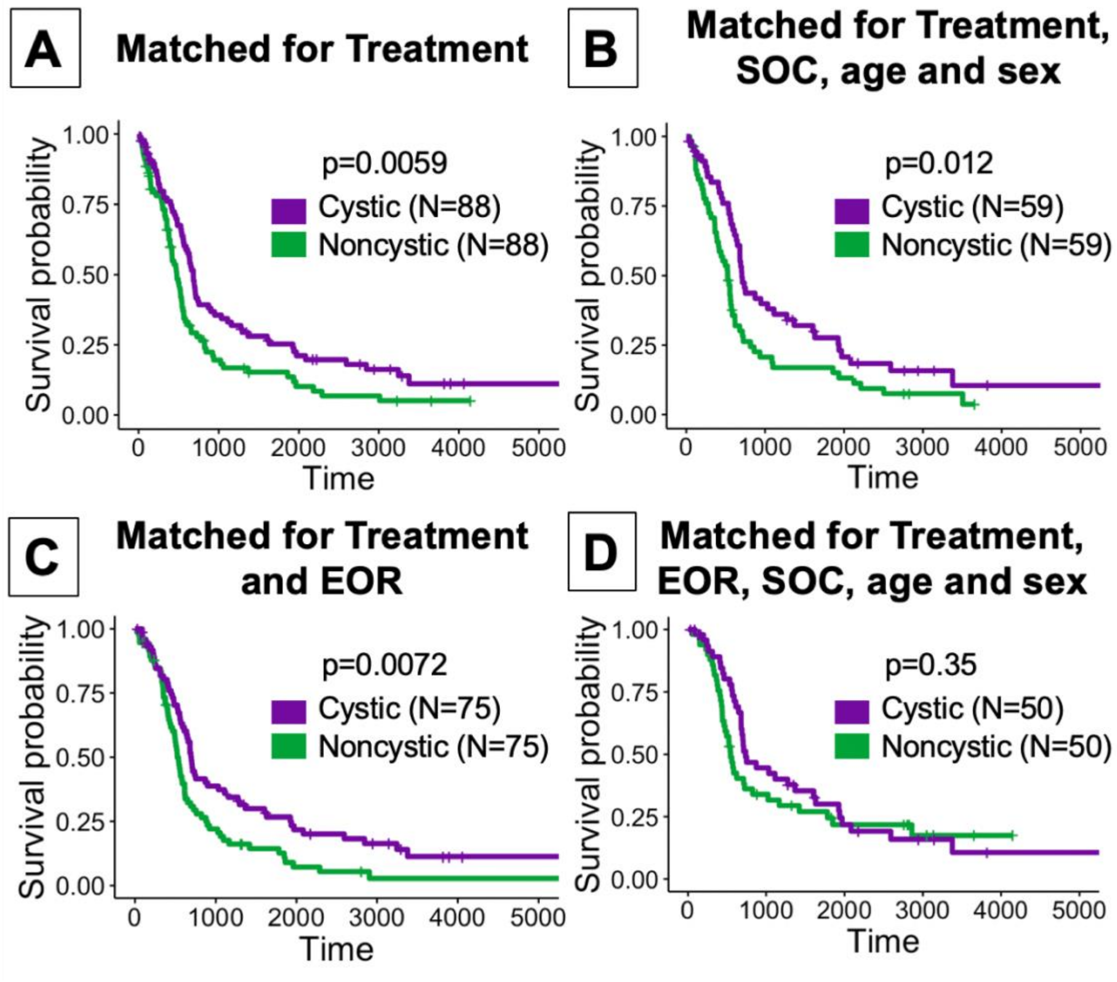

Example Kaplan-Meier plots for matched cohorts Using propensity score matching in RStudio, patients with noncystic GBM were matched with those with cystic GBM for **(A)** the top 10 most common known treatments received (see **Supplement 1**), **(B)** the top 10 most common known treatments received alongside diagnosis age, SOC status (received current SOC or did not) and patient sex, **(C)** the top 10 most common known treatments and extent of resection (EOR) and **(D)** the top 10 most common known treatments, EOR, SOC status, age at diagnosis and patient sex. **(A-C)** Within all of these matched cohort settings, we observed some significant differences in overall survival (OS). As the matching process is not unique, we ran 1000 cases to test for the consistency of these observations. In the setting of matched treatment only, 88.8% of matched cases (with 1000 unique matched combinations, mean p=0.024) resulted in significant differences in OS. In the case of matched treatment and age, sex and SOC status, 100% of matched cases were significant (with 2 unique matched combinations, mean p=0.012). For matched treatment and EOR, 48.2% were significant (with 1000 unique matched combinations, mean p=0.067). **(D)** We did not observe significance in this reduced cohort setting, with only 1 unique match with p=0.35.

SUPPLEMENT 3: Age-adjusted rate of cystic GBM:
Since the incidence of GBM varies with age, we used an age-adjusted rate of cyst presentation in GBM to assess whether presenting with cysts was more common in a particular age group. Age-adjusted rates are calculated by dividing the number of cystic GBM patients in a particular age groups in our cohort by the number of GBM patients in that age group in a larger "standard population". We used the 2011-2015 CBTRUS GBM data as our "standard population".

Age Group	Number of cystic GBM (n=87)*	CBTRUS count** (n=8665)	Age-adjusted SP rate (CBTRUS)***
20-34	10	255.26	0.392
35-44	11	558.24	0.197
45-54	20	1314.6	0.152
55-64	26	1928.9	0.135
65-74	15	2355.8	0.0637
75-84	3	1863.2	0.0161
85+	2	386.31	0.0518

*Patients diagnosed at an age younger than 20 years old were not included in this analysis
**The number of GBM patients in each age group was calculated from the "age-adjusted incidence rate" of GBM and 2000 US standard population counts provided in the CBTRUS 2011-2015 report.
***CBTRUS age-adjusted SP rates are represented as SP cases per 10 GB patients (raw rate x 10)

SUPPLEMENT 4: Other t-tests

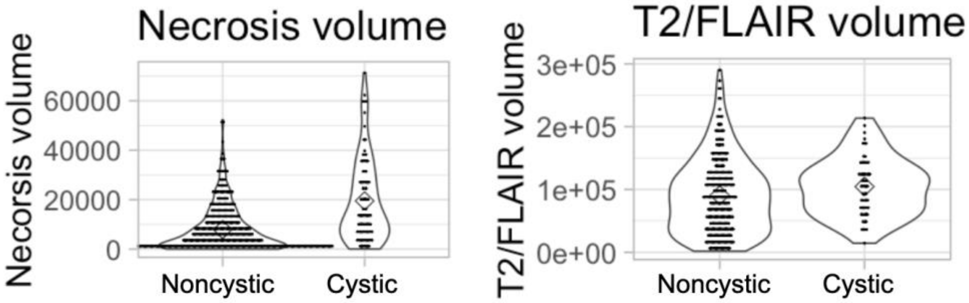

Comparison of cystic versus noncystic GBM patients for necrosis volume (inclusive of both central necrosis and cystic volumes) ($p<0.0001$) and T2/FLAIR volume ($p=0.053$)

SUPPLEMENT 5: T-tests for current SOC patients only

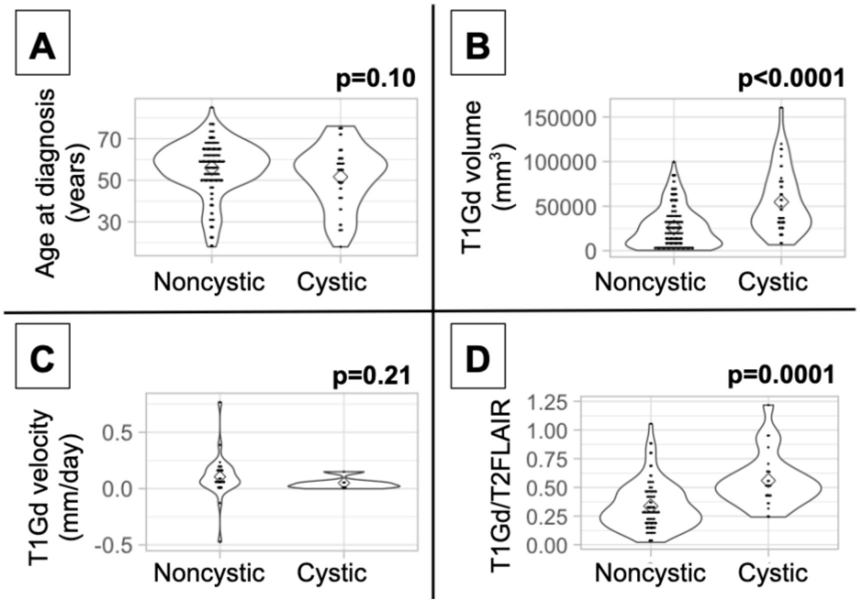

T-test comparison of **(A)** age at diagnosis, **(B)** T1Gd volume, **(C)** pre-surgical T1Gd growth velocity, and **(D)** the ratio of the T1Gd and T2FLAIR volumes (T1Gd/T2FLAIR) for cystic versus noncystic current SOC patients

SUPPLEMENT 6: Additional cystic vs noncystic GBM survival comparisons

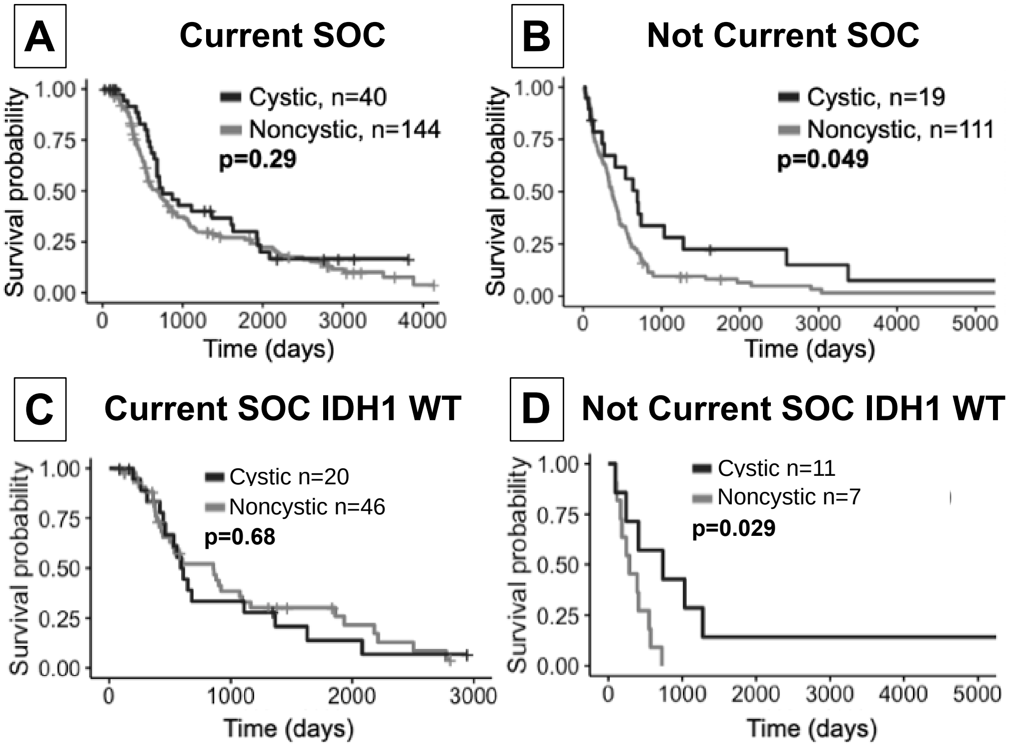

(A) Survival comparison for cystic vs noncystic GBM patients among current standard of care patients (n=184). **(B)** Patients who did not receive standard of care (n=130). Cystic patients had significantly improved survival compared to noncystic patients only among not current SOC patients (p=0.049). **(C)** Cystic vs noncsystic GBM patients who received the current standard of care with known IDH1 Wild Type (WT). We did not observe a survival benefit for cystic GBM (p=0.68). **(D)** We observed a significant survival benefit for IDH1 WT cystic GBM against IDH1 WT noncystic GBM in patients who did not receive the current standard of care (p=0.029).

SUPPLEMENT 7: Sex differences

Females

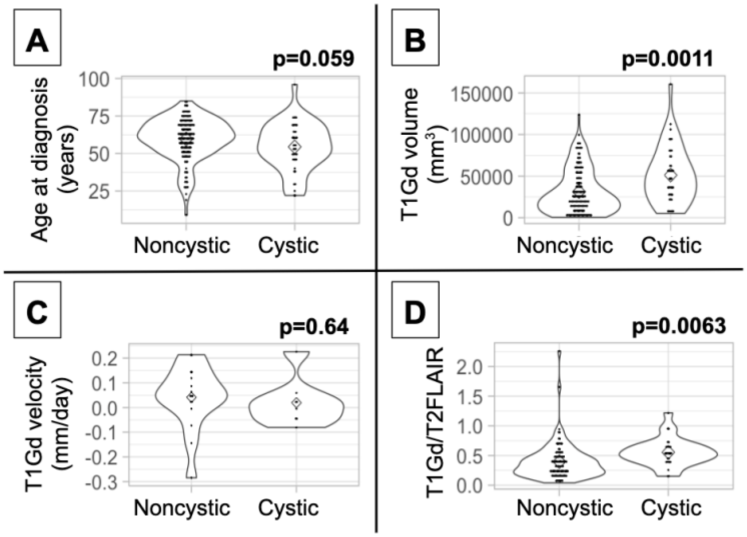

T-test comparison of (A) age at diagnosis, (B) T1Gd volume, (C) pre-surgical T1Gd growth velocity, and (D) the ratio of the T1Gd and T2FLAIR volumes (T1Gd/T2FLAIR) for cystic versus noncystic female patients

Males

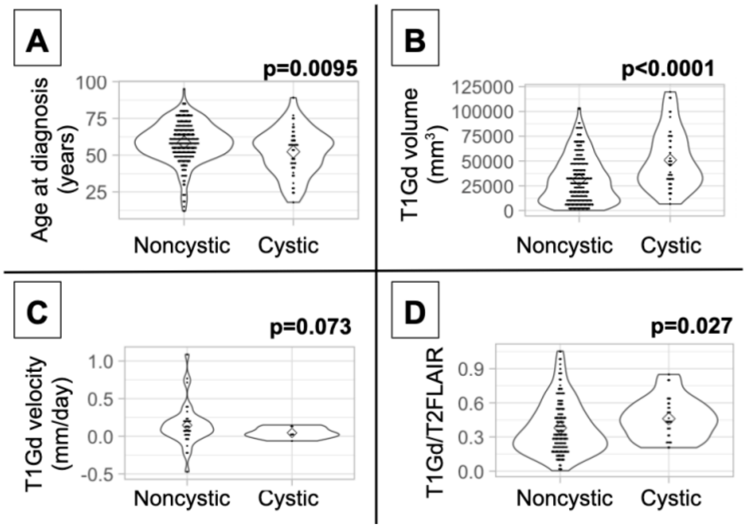

T-test comparison of (A) age at diagnosis, (B) T1Gd volume, (C) pre-surgical T1Gd growth velocity, and (D) the ratio of the T1Gd and T2FLAIR volumes (T1Gd/T2FLAIR) for cystic versus noncystic male patients

Multivariate CPH

	Females (n=190)		Males (n=303)	
	HR	p-value	HR	p-value
Age at diagnosis	1.03	**p<0.0001**	1.03	**p<0.0001**
Cystic GBM (yes = 1)	0.866	p=0.461	0.655	**p=0.0227**

Multivariate CPH of presence of cyst and age at diagnosis showing that presence of cystic was significantly associated for overall survival independent of age among males only.

	Females (n=135)		Males (n=215)	
	HR	p-value	HR	p-value
Age at diagnosis	1.03	**p<0.0001**	1.03	**p<0.0001**
Cystic GBM (yes = 1)	0.818	p=0.377	0.678	p=0.0548
EOR (biopsy = 1, STR = 2, GTR = 3)	0.656	**p=0.0014**	0.696	**p=0.0003**
Treatment protocol (current SOC = 1)	0.502	**p=0.0009**	0.600	**p=0.0008**

Multivariate CPH of presence of cyst, age at diagnosis, EOR, and treatment protocol. Presence of cystic approached significance for being independently associated with overall survival independent of other factors among males only.
